# *catena*-Poly[[copper(I)-bis[μ-bis(di­phenyl­phos­phino)methane-κ^2^ *P*:*P*′]-copper(I)-μ-2,2′-bipyridine-κ^2^ *N*:*N*′] bis(tetra­fluorido­borate) dichloromethane 2.5-solvate]

**DOI:** 10.1107/S1600536808001530

**Published:** 2008-01-23

**Authors:** Juan Mo, Gong-Zheng Hu, Wen Chen, Li Yuan, Yu-Shan Pan

**Affiliations:** a College of Animal Husbandry and Veterinary Studies, Henan Agricultural University, Zhengzhou, Henan Province 450002, People’s Republic of China

## Abstract

The title complex, {[Cu_2_(C_10_H_8_N_2_)(C_25_H_22_P_2_)_2_](BF_4_)_2_·2.5CH_2_Cl_2_}_*n*_, contains chains of Cu^I^ centres bridged alternately by two (diphenyl­phosphino)methane (dppm) and 4,4′-bipyridine (bpy) ligands. Each Cu^I^ atom is coordinated by one N atom of 4,4′-bipyridine (bpy) and two P atoms of two (diphenyl­phosphino)methane (dppm) ligand, and has a trigonal-planar coordination geometry. There is an inversion centre midway between each pair of adjacent Cu atoms. The distance of two Cu^I^ atoms separated by two (diphenyl­phosphino)methane bridging ligands is 3.732 (3) Å, and 4,4′-bipyridine 11.138 (5) Å.

## Related literature

For related literature, see: Ahuja *et al.* (2007 ▶); Liu *et al.* (2006 ▶); Park *et al.* (2001 ▶); Sekabunga *et al.* (2002 ▶); Yam *et al.* (2001 ▶).
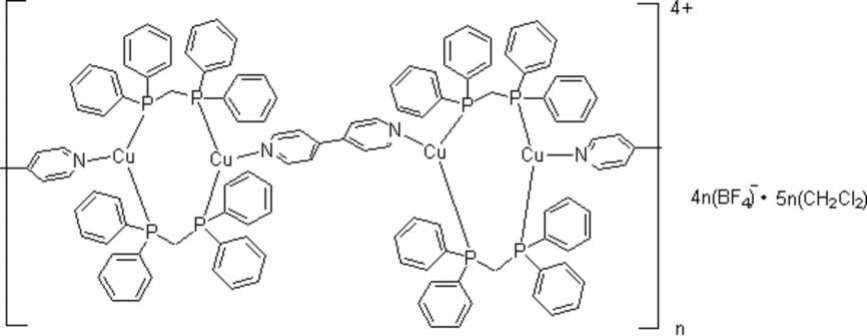


## Experimental

### 

#### Crystal data

[Cu_2_(C_10_H_8_N_2_)(C_25_H_22_P_2_)_2_](BF_4_)_2_·2.5CH_2_Cl_2_
*M* *_r_* = 1437.93
Triclinic, 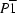

*a* = 10.9860 (12) Å
*b* = 16.1541 (18) Å
*c* = 20.336 (2) Å
α = 82.821 (2)°
β = 89.030 (2)°
γ = 74.237 (2)°
*V* = 3445.5 (7) Å^3^
*Z* = 2
Mo *K*α radiation
μ = 0.96 mm^−1^
*T* = 294 (2) K
0.22 × 0.12 × 0.06 mm

#### Data collection

Bruker SMART CCD area-detector diffractometer
Absorption correction: multi-scan (*SADABS*; Sheldrick, 1996 ▶) *T*_min_ = 0.871, *T*_max_ = 0.947
17673 measured reflections
12068 independent reflections
7620 reflections with *I* > 2σ(*I*)
*R*_int_ = 0.029

#### Refinement

*R*[*F* ^2^ > 2σ(*F* ^2^)] = 0.068
*wR*(*F* ^2^) = 0.231
*S* = 1.16
12068 reflections
802 parameters
142 restraints
H-atom parameters constrained
Δρ_max_ = 1.14 e Å^−3^
Δρ_min_ = −0.54 e Å^−3^

### 

Data collection: *SMART* (Bruker, 2000 ▶); cell refinement: *SAINT* (Bruker, 2000 ▶); data reduction: *SAINT*; program(s) used to solve structure: *SHELXTL* (Sheldrick, 2008 ▶); program(s) used to refine structure: *SHELXTL*; molecular graphics: *SHELXTL*; software used to prepare material for publication: *SHELXTL*.

## Supplementary Material

Crystal structure: contains datablocks global, I. DOI: 10.1107/S1600536808001530/hg2367sup1.cif

Structure factors: contains datablocks I. DOI: 10.1107/S1600536808001530/hg2367Isup2.hkl

Additional supplementary materials:  crystallographic information; 3D view; checkCIF report

## Figures and Tables

**Table:** 

Cu1—N1	2.018 (5)
Cu1—P3	2.2329 (18)
Cu1—P1	2.2515 (18)
Cu2—N2	2.022 (5)
Cu2—P2	2.2397 (19)
Cu2—P4	2.2505 (19)

**Table:** 

N1—Cu1—P3	118.66 (17)
N1—Cu1—P1	115.59 (17)
P3—Cu1—P1	122.10 (7)
N2—Cu2—P2	116.50 (18)
N2—Cu2—P4	111.90 (18)
P2—Cu2—P4	129.66 (7)

**Fig. 1.**
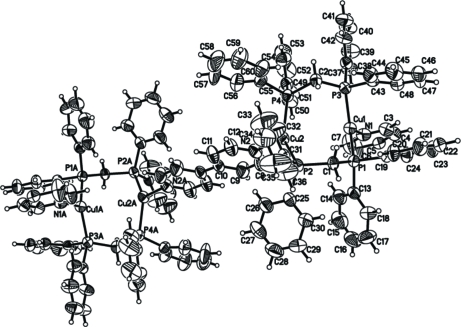
Molecular structure of the title compound showing the atom-numbering scheme and displacement ellipsoids drawn at the 50% probability level. The symmetry code for the unlabelled atoms is (-x, y, 0.5 - z). Free (BF4)- anions and dichloromethane solvent molecules are not shown.

## supplementary crystallographic information

### Comment

4,4'-bipyridine is widely used in the construction of transition metal coordination complexes, because of its rod-like shape, which allows the ligand to connect metal ions into an extended array (Park *et al.*, 2001). The hydrocarbon backbone bisphosphine ligand Ph_2_PCH_2_PPh_2_ (dppm) has been widely studied and has shown great versatility as a ligand for its ability to bridge metal centers in the µ-bonding mode, forming bi- and polynuclear complexes (Ahuja *et al.*, 2007; Sekabunga *et al.*, 2002; Yam *et al.*, 2001). Here, we report crystal structure of the title compound, (I), one-dimensional chain coordination polymer bridged by (diphenylphosphino)methane and 4,4'-bipyridine.

The Cu atom in (I) (Fig. 1) has a trigonal–planar coordination geometry involving the N atom of the 4,4'-bipyridine ligand and two P atoms of two (diphenylphosphino)methane ligands. A four-coordinate tetrahedral coordination sphere is more usual for Cu^I^ atoms and the trigonal–planar arrangement in the presen complex may reflect the large size of the rigid organic ligands which would spacially hinder any additional donor atoms from entering the metal coordination sphere. The Cu—N bonds lengths are 2.018 (5) and 2.022 (5) Å, while the Cu—P bonds are between 2.233 (2) and 2.252 (2) Å (Table 1). The two Cu^I^ ions in (I) are doubly bridged by two (diphenylphosphino)methane ligands and single bridged by 4,4'-bipyridine ligands. The Cu···Cu distance bridged by two (diphenylphosphino)methane is 3.732 (3) Å, much shorter than reported data, 4.720 (5)Å (Liu *et al.*, 2006).

### Experimental

A 10 ml dichloromethane solution of 4,4'-bipyridine (0.078 g, 0.5 mmol) was added to a 20 mL dichloromethane solution of [Cu(CH_3_CN)_4_][BF_4_] (0.372 g, 1.0 mmol) and (diphenylphosphino)methane (0.384 g, 1.0 mmol) under N_2_ atmosphere. The mixture was stirred for 10 h. Yellow crystals suitable for X-ray diffraction were formed by vapour diffusion of diethyl ethyl ether into dichloromethane solution.

### Refinement

All hydrogen atoms were generated geometrically (C—H bond lengths fixed at 0.93 Å), assigned appropriated isotropic thermal parameters, *U*_iso_(H) = 1.2*U*_eq_(C). During the structure refinement, a region of electron density was identified as a disordered dichloromethane solvent molecule. The site occupancies were determined using an isotropic model using *DFIX* 1.75 (1) (C61—Cl1, C61—Cl2, C61—Cl1', C61—Cl2') Å restraints in subsequent refinement cycles. 'ISOR' and 'simu' restraint was applied for atoms from F1 to Cl6 for preventing these atoms from becoming 'non-positive-definite'.

### Crystal data

**Table:**

[Cu_2_(C_10_H_8_N_2_)(C_25_H_22_P_2_)_2_](BF_4_)_2_·2.5CH_2_Cl_2_	*Z* = 2
*M**_r_* = 1437.93	*F*_000_ = 1462
Triclinic, *P*1	*D*_x_ = 1.386 Mg m^−^^3^
Hall symbol: -P 1	Mo *K*α radiation λ = 0.71073 Å
*a* = 10.9860 (12) Å	Cell parameters from 4736 reflections
*b* = 16.1541 (18) Å	θ = 2.4–23.6º
*c* = 20.336 (2) Å	µ = 0.96 mm^−^^1^
α = 82.821 (2)º	*T* = 294 (2) K
β = 89.030 (2)º	Block, yellow
γ = 74.237 (2)º	0.22 × 0.12 × 0.06 mm
*V* = 3445.5 (7) Å^3^	

### Data collection

**Table:**

Bruker SMART CCD area-detector diffractometer	12068 independent reflections
Radiation source: fine-focus sealed tube	7620 reflections with *I* > 2σ(*I*)
Monochromator: graphite	*R*_int_ = 0.029
*T* = 294(2) K	θ_max_ = 25.0º
phi and ω scans	θ_min_ = 1.0º
Absorption correction: multi-scan(SADABS; Sheldrick, 1996)	*h* = −13→13
*T*_min_ = 0.871, *T*_max_ = 0.947	*k* = −19→17
17673 measured reflections	*l* = −24→22

### Refinement

**Table:**

Refinement on *F*^2^	Secondary atom site location: difference Fourier map
Least-squares matrix: full	Hydrogen site location: inferred from neighbouring sites
*R*[*F*^2^ > 2σ(*F*^2^)] = 0.068	H-atom parameters constrained
*wR*(*F*^2^) = 0.231	*w* = 1/[σ^2^(*F*_o_^2^) + (0.1056*P*)^2^ + 4.6434*P*] where *P* = (*F*_o_^2^ + 2*F*_c_^2^)/3
*S* = 1.16	(Δ/σ)_max_ = 0.005
12068 reflections	Δρ_max_ = 1.14 e Å^−^^3^
802 parameters	Δρ_min_ = −0.54 e Å^−^^3^
142 restraints	Extinction correction: none
Primary atom site location: structure-invariant direct methods	

### Special details

**Table:**

Geometry. All e.s.d.'s (except the e.s.d. in the dihedral angle between two l.s. planes) are estimated using the full covariance matrix. The cell e.s.d.'s are taken into account individually in the estimation of e.s.d.'s in distances, angles and torsion angles; correlations between e.s.d.'s in cell parameters are only used when they are defined by crystal symmetry. An approximate (isotropic) treatment of cell e.s.d.'s is used for estimating e.s.d.'s involving l.s. planes.
Refinement. Refinement of *F*^2^ against ALL reflections. The weighted *R*-factor *wR* and goodness of fit *S* are based on *F*^2^, conventional *R*-factors *R* are based on *F*, with *F* set to zero for negative *F*^2^. The threshold expression of *F*^2^ > σ(*F*^2^) is used only for calculating *R*-factors(gt) *etc*. and is not relevant to the choice of reflections for refinement. *R*-factors based on *F*^2^ are statistically about twice as large as those based on *F*, and *R*- factors based on ALL data will be even larger.

### Fractional atomic coordinates and isotropic or equivalent isotropic displacement parameters (Å^2^)

**Table:**

	*x*	*y*	*z*	*U*_iso_*/*U*_eq_	Occ. (<1)
Cu1	0.78585 (7)	0.65849 (5)	0.13999 (4)	0.0383 (2)	
Cu2	0.78806 (7)	0.65568 (5)	0.32279 (4)	0.0428 (2)	
P1	0.98370 (15)	0.57875 (11)	0.16926 (8)	0.0355 (4)	
P2	0.99714 (16)	0.59589 (11)	0.32001 (8)	0.0389 (4)	
P3	0.72735 (15)	0.80272 (10)	0.13569 (8)	0.0366 (4)	
P4	0.67290 (16)	0.78988 (11)	0.28283 (8)	0.0395 (4)	
N1	0.6855 (5)	0.6018 (4)	0.0866 (3)	0.0495 (14)	
N2	0.6907 (5)	0.5954 (4)	0.3888 (3)	0.0525 (15)	
C1	1.0679 (6)	0.6038 (4)	0.2380 (3)	0.0375 (14)	
H1A	1.1528	0.5650	0.2409	0.045*	
H1B	1.0757	0.6623	0.2271	0.045*	
C2	0.7395 (6)	0.8469 (4)	0.2137 (3)	0.0425 (15)	
H2A	0.8277	0.8415	0.2235	0.051*	
H2B	0.6947	0.9081	0.2087	0.051*	
C3	0.6815 (7)	0.6091 (5)	0.0209 (4)	0.0549 (19)	
H3	0.7289	0.6421	−0.0028	0.066*	
C4	0.6106 (7)	0.5704 (5)	−0.0141 (4)	0.0532 (18)	
H4	0.6118	0.5775	−0.0602	0.064*	
C5	0.5390 (6)	0.5220 (4)	0.0178 (3)	0.0426 (15)	
C6	0.5439 (9)	0.5137 (7)	0.0860 (4)	0.084 (3)	
H6	0.4981	0.4804	0.1106	0.101*	
C7	0.6158 (9)	0.5541 (6)	0.1177 (4)	0.075 (3)	
H7	0.6158	0.5479	0.1638	0.090*	
C8	0.6417 (9)	0.5347 (6)	0.3733 (4)	0.070 (2)	
H8	0.6570	0.5173	0.3313	0.084*	
C9	0.5684 (9)	0.4951 (6)	0.4156 (4)	0.072 (2)	
H9	0.5372	0.4519	0.4019	0.087*	
C10	0.5414 (6)	0.5194 (5)	0.4778 (3)	0.0473 (17)	
C11	0.5956 (8)	0.5812 (6)	0.4941 (4)	0.064 (2)	
H11	0.5836	0.5987	0.5361	0.077*	
C12	0.6671 (7)	0.6179 (6)	0.4498 (4)	0.062 (2)	
H12	0.7008	0.6604	0.4627	0.074*	
C13	1.0142 (7)	0.4618 (4)	0.1806 (3)	0.0445 (16)	
C14	0.9186 (8)	0.4251 (5)	0.2047 (4)	0.061 (2)	
H14	0.8407	0.4606	0.2153	0.073*	
C15	0.9390 (12)	0.3367 (7)	0.2129 (5)	0.086 (3)	
H15	0.8738	0.3131	0.2282	0.103*	
C16	1.0505 (14)	0.2838 (6)	0.1992 (5)	0.093 (3)	
H16	1.0623	0.2241	0.2056	0.112*	
C17	1.1473 (11)	0.3168 (6)	0.1760 (5)	0.092 (3)	
H17	1.2249	0.2797	0.1669	0.110*	
C18	1.1293 (8)	0.4070 (5)	0.1658 (4)	0.063 (2)	
H18	1.1943	0.4299	0.1491	0.076*	
C19	1.0747 (6)	0.6024 (4)	0.0971 (3)	0.0398 (15)	
C20	1.1738 (7)	0.6396 (5)	0.0979 (4)	0.0550 (19)	
H20	1.2033	0.6488	0.1382	0.066*	
C21	1.2309 (9)	0.6638 (5)	0.0393 (4)	0.071 (2)	
H21	1.2968	0.6896	0.0407	0.085*	
C22	1.1886 (9)	0.6493 (5)	−0.0210 (4)	0.067 (2)	
H22	1.2259	0.6651	−0.0602	0.081*	
C23	1.0928 (9)	0.6119 (6)	−0.0220 (4)	0.068 (2)	
H23	1.0648	0.6016	−0.0624	0.081*	
C24	1.0350 (7)	0.5885 (5)	0.0361 (3)	0.0534 (18)	
H24	0.9688	0.5632	0.0340	0.064*	
C25	1.0569 (7)	0.4842 (4)	0.3587 (3)	0.0458 (16)	
C26	0.9971 (8)	0.4582 (6)	0.4146 (4)	0.068 (2)	
H26	0.9282	0.4977	0.4305	0.081*	
C27	1.0364 (10)	0.3756 (6)	0.4474 (4)	0.082 (3)	
H27	0.9945	0.3594	0.4849	0.099*	
C28	1.1388 (11)	0.3170 (6)	0.4240 (5)	0.084 (3)	
H28	1.1665	0.2610	0.4460	0.100*	
C29	1.1986 (10)	0.3405 (6)	0.3695 (5)	0.089 (3)	
H29	1.2669	0.3005	0.3538	0.107*	
C30	1.1588 (8)	0.4248 (5)	0.3364 (4)	0.067 (2)	
H30	1.2015	0.4406	0.2991	0.081*	
C31	1.0784 (7)	0.6594 (4)	0.3634 (3)	0.0447 (16)	
C32	1.0151 (10)	0.7392 (6)	0.3748 (6)	0.098 (4)	
H32	0.9295	0.7595	0.3636	0.117*	
C33	1.0740 (12)	0.7925 (7)	0.4027 (7)	0.121 (5)	
H33	1.0285	0.8486	0.4087	0.145*	
C34	1.1969 (11)	0.7635 (7)	0.4213 (5)	0.094 (3)	
H34	1.2365	0.7982	0.4413	0.113*	
C35	1.2608 (11)	0.6829 (8)	0.4102 (7)	0.131 (5)	
H35	1.3458	0.6619	0.4225	0.157*	
C36	1.2027 (9)	0.6315 (6)	0.3812 (6)	0.097 (4)	
H36	1.2493	0.5763	0.3736	0.117*	
C37	0.5734 (6)	0.8662 (4)	0.1014 (3)	0.0441 (16)	
C38	0.4900 (7)	0.8261 (5)	0.0793 (4)	0.0571 (19)	
H38	0.5128	0.7659	0.0819	0.069*	
C39	0.3730 (8)	0.8729 (6)	0.0533 (5)	0.073 (2)	
H39	0.3181	0.8444	0.0382	0.088*	
C40	0.3380 (8)	0.9606 (6)	0.0498 (5)	0.073 (2)	
H40	0.2591	0.9922	0.0323	0.087*	
C41	0.4191 (8)	1.0023 (5)	0.0721 (4)	0.071 (2)	
H41	0.3946	1.0624	0.0702	0.086*	
C42	0.5378 (7)	0.9556 (5)	0.0976 (4)	0.059 (2)	
H42	0.5931	0.9844	0.1121	0.071*	
C43	0.8411 (6)	0.8383 (4)	0.0809 (3)	0.0398 (15)	
C44	0.9262 (7)	0.8800 (5)	0.1004 (4)	0.0577 (19)	
H44	0.9246	0.8949	0.1431	0.069*	
C45	1.0150 (8)	0.8998 (6)	0.0553 (5)	0.079 (3)	
H45	1.0732	0.9269	0.0688	0.094*	
C46	1.0176 (8)	0.8800 (6)	−0.0076 (5)	0.072 (3)	
H46	1.0762	0.8945	−0.0372	0.086*	
C47	0.9345 (9)	0.8392 (5)	−0.0273 (4)	0.070 (2)	
H47	0.9370	0.8250	−0.0703	0.084*	
C48	0.8463 (7)	0.8185 (5)	0.0161 (4)	0.0553 (19)	
H48	0.7894	0.7909	0.0017	0.066*	
C49	0.5100 (6)	0.8011 (5)	0.2594 (3)	0.0461 (16)	
C50	0.4726 (8)	0.7277 (5)	0.2510 (4)	0.065 (2)	
H50	0.5312	0.6736	0.2573	0.078*	
C51	0.3485 (9)	0.7345 (7)	0.2334 (5)	0.091 (3)	
H51	0.3243	0.6846	0.2288	0.109*	
C52	0.2625 (9)	0.8124 (8)	0.2227 (5)	0.093 (3)	
H52	0.1804	0.8161	0.2096	0.112*	
C53	0.2958 (8)	0.8862 (7)	0.2313 (5)	0.085 (3)	
H53	0.2357	0.9397	0.2249	0.102*	
C54	0.4209 (7)	0.8813 (5)	0.2496 (4)	0.063 (2)	
H54	0.4436	0.9314	0.2552	0.076*	
C55	0.6621 (7)	0.8577 (5)	0.3482 (3)	0.0506 (17)	
C56	0.5872 (9)	0.8459 (6)	0.4022 (4)	0.082 (3)	
H56	0.5355	0.8089	0.4023	0.098*	
C57	0.5906 (13)	0.8909 (9)	0.4570 (5)	0.118 (4)	
H57	0.5426	0.8820	0.4940	0.142*	
C58	0.6634 (15)	0.9473 (9)	0.4567 (6)	0.117 (4)	
H58	0.6628	0.9774	0.4929	0.141*	
C59	0.7358 (11)	0.9597 (8)	0.4046 (6)	0.100 (3)	
H59	0.7848	0.9985	0.4045	0.120*	
C60	0.7373 (8)	0.9144 (6)	0.3511 (4)	0.070 (2)	
H60	0.7900	0.9220	0.3159	0.084*	
B1	0.9148 (14)	0.0567 (12)	0.2166 (8)	0.103 (5)	
B2	0.4736 (11)	0.4350 (9)	0.2520 (6)	0.081 (3)	
F1	0.9614 (9)	−0.0327 (6)	0.2453 (5)	0.168 (3)	
F2	1.0190 (10)	0.0665 (7)	0.1914 (5)	0.187 (4)	
F3	0.8700 (9)	0.1076 (7)	0.2601 (5)	0.186 (4)	
F4	0.8255 (10)	0.0519 (7)	0.1752 (5)	0.196 (4)	
F5	0.5923 (7)	0.4249 (6)	0.2422 (4)	0.146 (3)	
F6	0.4082 (9)	0.5169 (6)	0.2445 (5)	0.177 (3)	
F7	0.4209 (9)	0.4062 (6)	0.2021 (4)	0.167 (3)	
F8	0.4520 (7)	0.3947 (5)	0.3116 (3)	0.133 (2)	
C61	0.8862 (11)	0.9237 (16)	0.6448 (6)	0.123 (8)	0.25
H61A	0.9490	0.8679	0.6511	0.148*	0.25
H61B	0.8997	0.9561	0.6798	0.148*	0.25
Cl1	0.9139 (14)	0.9790 (8)	0.5688 (5)	0.088 (4)	0.25
Cl2	0.7380 (10)	0.9060 (8)	0.6547 (9)	0.085 (4)	0.25
C61'	0.8862 (11)	0.9237 (16)	0.6448 (6)	0.123 (8)	0.25
H61C	0.9049	0.9650	0.6711	0.148*	0.25
H61D	0.9262	0.8660	0.6667	0.148*	0.25
Cl1'	0.9515 (13)	0.9359 (9)	0.5672 (5)	0.088 (4)	0.25
Cl2'	0.7237 (10)	0.9386 (11)	0.6427 (8)	0.093 (5)	0.25
C62	0.2991 (13)	0.7668 (9)	0.6418 (7)	0.129 (4)	
H62A	0.2551	0.8206	0.6583	0.155*	
H62B	0.3513	0.7293	0.6776	0.155*	
Cl3	0.3935 (6)	0.7879 (4)	0.5765 (3)	0.198 (2)	
Cl4	0.1885 (5)	0.7164 (4)	0.6152 (2)	0.1833 (19)	
C63	0.4294 (14)	0.8051 (9)	0.8138 (7)	0.135 (5)	
H63A	0.4437	0.7503	0.7961	0.162*	
H63B	0.3401	0.8352	0.8086	0.162*	
Cl5	0.5165 (7)	0.8663 (4)	0.7710 (3)	0.227 (3)	
Cl6	0.4723 (6)	0.7864 (3)	0.8974 (2)	0.192 (2)	

### Atomic displacement parameters (Å^2^)

**Table:**

	*U*^11^	*U*^22^	*U*^33^	*U*^12^	*U*^13^	*U*^23^
Cu1	0.0361 (4)	0.0390 (4)	0.0439 (5)	−0.0160 (3)	−0.0046 (3)	−0.0070 (3)
Cu2	0.0386 (5)	0.0483 (5)	0.0426 (5)	−0.0170 (4)	0.0059 (3)	0.0012 (4)
P1	0.0330 (8)	0.0404 (9)	0.0350 (9)	−0.0125 (7)	−0.0017 (6)	−0.0068 (7)
P2	0.0376 (9)	0.0466 (10)	0.0329 (9)	−0.0138 (8)	0.0007 (7)	−0.0011 (7)
P3	0.0380 (9)	0.0384 (9)	0.0364 (9)	−0.0165 (7)	−0.0018 (7)	−0.0025 (7)
P4	0.0374 (9)	0.0428 (9)	0.0404 (9)	−0.0148 (7)	0.0052 (7)	−0.0047 (7)
N1	0.048 (3)	0.045 (3)	0.060 (4)	−0.016 (3)	−0.012 (3)	−0.011 (3)
N2	0.048 (3)	0.059 (4)	0.054 (4)	−0.025 (3)	0.007 (3)	0.006 (3)
C1	0.035 (3)	0.048 (4)	0.031 (3)	−0.015 (3)	−0.001 (3)	−0.006 (3)
C2	0.049 (4)	0.045 (4)	0.040 (4)	−0.024 (3)	0.005 (3)	−0.008 (3)
C3	0.054 (4)	0.053 (4)	0.063 (5)	−0.024 (4)	−0.011 (4)	−0.004 (4)
C4	0.057 (5)	0.057 (4)	0.052 (4)	−0.025 (4)	−0.011 (3)	−0.008 (3)
C5	0.032 (3)	0.046 (4)	0.052 (4)	−0.013 (3)	−0.006 (3)	−0.009 (3)
C6	0.101 (7)	0.136 (9)	0.054 (5)	−0.096 (7)	0.003 (5)	−0.018 (5)
C7	0.100 (7)	0.108 (7)	0.046 (5)	−0.074 (6)	−0.005 (4)	−0.017 (4)
C8	0.093 (7)	0.074 (6)	0.054 (5)	−0.041 (5)	0.028 (4)	−0.009 (4)
C9	0.097 (7)	0.069 (5)	0.066 (5)	−0.048 (5)	0.032 (5)	−0.013 (4)
C10	0.047 (4)	0.054 (4)	0.039 (4)	−0.017 (3)	0.006 (3)	0.006 (3)
C11	0.068 (5)	0.092 (6)	0.043 (4)	−0.045 (5)	0.005 (4)	0.001 (4)
C12	0.062 (5)	0.084 (6)	0.053 (5)	−0.043 (4)	0.003 (4)	−0.001 (4)
C13	0.052 (4)	0.042 (4)	0.040 (4)	−0.016 (3)	−0.006 (3)	−0.001 (3)
C14	0.074 (5)	0.059 (5)	0.056 (5)	−0.031 (4)	0.007 (4)	0.000 (4)
C15	0.129 (10)	0.069 (6)	0.075 (6)	−0.058 (7)	0.003 (6)	0.002 (5)
C16	0.157 (12)	0.048 (6)	0.077 (7)	−0.036 (7)	−0.003 (7)	0.001 (5)
C17	0.113 (9)	0.057 (6)	0.091 (7)	0.008 (6)	−0.009 (6)	−0.022 (5)
C18	0.061 (5)	0.051 (5)	0.073 (5)	−0.007 (4)	−0.005 (4)	−0.004 (4)
C19	0.044 (4)	0.036 (3)	0.036 (4)	−0.006 (3)	0.005 (3)	−0.004 (3)
C20	0.070 (5)	0.065 (5)	0.044 (4)	−0.039 (4)	0.011 (4)	−0.017 (3)
C21	0.083 (6)	0.070 (6)	0.073 (6)	−0.042 (5)	0.027 (5)	−0.014 (4)
C22	0.084 (6)	0.054 (5)	0.054 (5)	−0.007 (4)	0.021 (4)	0.002 (4)
C23	0.080 (6)	0.083 (6)	0.036 (4)	−0.011 (5)	0.003 (4)	−0.014 (4)
C24	0.050 (4)	0.071 (5)	0.039 (4)	−0.015 (4)	−0.001 (3)	−0.011 (3)
C25	0.049 (4)	0.052 (4)	0.038 (4)	−0.018 (3)	−0.005 (3)	−0.001 (3)
C26	0.073 (6)	0.068 (5)	0.055 (5)	−0.017 (4)	0.006 (4)	0.010 (4)
C27	0.102 (8)	0.081 (7)	0.065 (6)	−0.039 (6)	0.002 (5)	0.023 (5)
C28	0.116 (9)	0.051 (5)	0.079 (7)	−0.022 (6)	−0.020 (6)	0.010 (5)
C29	0.103 (8)	0.055 (5)	0.086 (7)	0.014 (5)	0.002 (6)	−0.003 (5)
C30	0.068 (5)	0.061 (5)	0.061 (5)	−0.002 (4)	0.004 (4)	0.004 (4)
C31	0.052 (4)	0.051 (4)	0.032 (3)	−0.017 (3)	−0.005 (3)	−0.003 (3)
C32	0.073 (6)	0.080 (7)	0.147 (10)	−0.014 (5)	−0.036 (6)	−0.051 (7)
C33	0.106 (9)	0.086 (8)	0.176 (13)	−0.013 (7)	−0.042 (9)	−0.064 (8)
C34	0.104 (8)	0.081 (7)	0.104 (8)	−0.029 (6)	−0.043 (6)	−0.020 (6)
C35	0.091 (8)	0.089 (8)	0.212 (15)	−0.014 (7)	−0.085 (9)	−0.030 (9)
C36	0.075 (7)	0.071 (6)	0.149 (10)	−0.015 (5)	−0.050 (6)	−0.027 (6)
C37	0.043 (4)	0.047 (4)	0.042 (4)	−0.014 (3)	0.001 (3)	−0.002 (3)
C38	0.045 (4)	0.053 (4)	0.073 (5)	−0.011 (4)	−0.011 (4)	−0.010 (4)
C39	0.049 (5)	0.069 (6)	0.101 (7)	−0.017 (4)	−0.019 (5)	−0.007 (5)
C40	0.044 (5)	0.067 (6)	0.097 (7)	−0.008 (4)	−0.008 (4)	0.013 (5)
C41	0.063 (5)	0.050 (5)	0.092 (6)	−0.007 (4)	−0.004 (5)	0.009 (4)
C42	0.060 (5)	0.044 (4)	0.072 (5)	−0.017 (4)	−0.015 (4)	0.009 (4)
C43	0.042 (4)	0.029 (3)	0.046 (4)	−0.008 (3)	0.003 (3)	−0.001 (3)
C44	0.053 (5)	0.062 (5)	0.063 (5)	−0.024 (4)	0.008 (4)	−0.007 (4)
C45	0.055 (5)	0.086 (6)	0.097 (7)	−0.035 (5)	0.017 (5)	0.009 (5)
C46	0.061 (5)	0.068 (6)	0.078 (6)	−0.013 (4)	0.031 (5)	0.007 (5)
C47	0.096 (7)	0.059 (5)	0.046 (5)	−0.008 (5)	0.024 (4)	−0.003 (4)
C48	0.064 (5)	0.049 (4)	0.052 (4)	−0.015 (4)	0.005 (4)	−0.007 (3)
C49	0.040 (4)	0.053 (4)	0.048 (4)	−0.020 (3)	0.008 (3)	−0.003 (3)
C50	0.059 (5)	0.065 (5)	0.074 (5)	−0.029 (4)	−0.005 (4)	0.004 (4)
C51	0.075 (7)	0.097 (8)	0.114 (8)	−0.056 (6)	−0.033 (6)	0.017 (6)
C52	0.056 (6)	0.114 (9)	0.117 (9)	−0.044 (6)	−0.017 (5)	0.009 (7)
C53	0.042 (5)	0.101 (8)	0.092 (7)	0.007 (5)	0.002 (4)	0.005 (6)
C54	0.048 (5)	0.062 (5)	0.079 (6)	−0.012 (4)	0.011 (4)	−0.010 (4)
C55	0.047 (4)	0.054 (4)	0.047 (4)	−0.004 (3)	0.002 (3)	−0.012 (3)
C56	0.091 (7)	0.091 (7)	0.062 (6)	−0.021 (6)	0.023 (5)	−0.017 (5)
C57	0.144 (12)	0.155 (12)	0.060 (7)	−0.037 (10)	0.045 (7)	−0.043 (7)
C58	0.151 (12)	0.121 (10)	0.081 (8)	−0.018 (9)	−0.011 (8)	−0.057 (8)
C59	0.118 (9)	0.114 (9)	0.083 (8)	−0.039 (7)	0.003 (7)	−0.051 (7)
C60	0.077 (6)	0.073 (6)	0.070 (6)	−0.027 (5)	0.003 (4)	−0.028 (5)
B1	0.081 (9)	0.138 (13)	0.104 (10)	−0.031 (9)	0.001 (8)	−0.061 (10)
B2	0.068 (7)	0.094 (9)	0.083 (8)	−0.035 (7)	−0.008 (6)	0.007 (7)
F1	0.171 (7)	0.155 (6)	0.176 (7)	−0.053 (6)	−0.006 (5)	0.007 (5)
F2	0.179 (7)	0.185 (7)	0.199 (7)	−0.067 (6)	0.055 (6)	−0.001 (6)
F3	0.178 (7)	0.203 (7)	0.173 (6)	−0.010 (6)	0.010 (5)	−0.111 (6)
F4	0.171 (7)	0.195 (7)	0.207 (7)	0.007 (6)	−0.057 (6)	−0.089 (6)
F5	0.083 (4)	0.202 (7)	0.138 (5)	−0.036 (4)	0.002 (4)	0.028 (5)
F6	0.183 (7)	0.121 (5)	0.192 (7)	0.006 (5)	0.040 (6)	0.006 (5)
F7	0.183 (7)	0.202 (7)	0.149 (6)	−0.106 (6)	−0.012 (5)	−0.025 (5)
F8	0.116 (5)	0.163 (6)	0.115 (5)	−0.055 (4)	−0.003 (4)	0.035 (4)
C61	0.125 (10)	0.131 (10)	0.115 (10)	−0.033 (7)	−0.010 (7)	−0.020 (7)
Cl1	0.110 (8)	0.085 (7)	0.088 (6)	−0.052 (6)	−0.004 (5)	−0.022 (5)
Cl2	0.102 (7)	0.055 (6)	0.092 (8)	−0.015 (5)	0.017 (6)	0.001 (5)
C61'	0.125 (10)	0.131 (10)	0.115 (10)	−0.033 (7)	−0.010 (7)	−0.020 (7)
Cl1'	0.086 (7)	0.102 (8)	0.071 (6)	−0.016 (6)	0.006 (5)	−0.017 (6)
Cl2'	0.098 (7)	0.107 (9)	0.065 (7)	−0.012 (6)	0.011 (5)	−0.019 (7)
C62	0.137 (8)	0.130 (8)	0.115 (8)	−0.018 (7)	0.011 (7)	−0.031 (7)
Cl3	0.212 (5)	0.187 (4)	0.187 (4)	−0.058 (4)	0.041 (4)	0.009 (3)
Cl4	0.197 (5)	0.200 (4)	0.160 (4)	−0.059 (4)	0.002 (3)	−0.035 (3)
C63	0.138 (8)	0.120 (8)	0.145 (9)	−0.028 (7)	−0.013 (7)	−0.020 (7)
Cl5	0.242 (6)	0.204 (5)	0.221 (5)	−0.055 (4)	0.047 (4)	0.005 (4)
Cl6	0.242 (5)	0.182 (4)	0.141 (3)	−0.044 (4)	−0.033 (3)	−0.007 (3)

### Geometric parameters (Å, °)

**Table:**

Cu1—N1	2.018 (5)	C30—H30	0.9300
Cu1—P3	2.2329 (18)	C31—C32	1.334 (11)
Cu1—P1	2.2515 (18)	C31—C36	1.356 (11)
Cu2—N2	2.022 (5)	C32—C33	1.387 (12)
Cu2—P2	2.2397 (19)	C32—H32	0.9300
Cu2—P4	2.2505 (19)	C33—C34	1.347 (14)
P1—C13	1.812 (7)	C33—H33	0.9300
P1—C19	1.821 (6)	C34—C35	1.345 (15)
P1—C1	1.834 (6)	C34—H34	0.9300
P2—C25	1.823 (7)	C35—C36	1.367 (13)
P2—C1	1.833 (6)	C35—H35	0.9300
P2—C31	1.838 (7)	C36—H36	0.9300
P3—C43	1.820 (6)	C37—C38	1.368 (10)
P3—C37	1.821 (7)	C37—C42	1.382 (10)
P3—C2	1.841 (6)	C38—C39	1.378 (10)
P4—C55	1.808 (7)	C38—H38	0.9300
P4—C49	1.815 (7)	C39—C40	1.355 (11)
P4—C2	1.835 (6)	C39—H39	0.9300
N1—C3	1.327 (9)	C40—C41	1.368 (12)
N1—C7	1.328 (9)	C40—H40	0.9300
N2—C8	1.312 (10)	C41—C42	1.388 (11)
N2—C12	1.336 (9)	C41—H41	0.9300
C1—H1A	0.9700	C42—H42	0.9300
C1—H1B	0.9700	C43—C44	1.381 (9)
C2—H2A	0.9700	C43—C48	1.392 (9)
C2—H2B	0.9700	C44—C45	1.399 (10)
C3—C4	1.377 (9)	C44—H44	0.9300
C3—H3	0.9300	C45—C46	1.356 (12)
C4—C5	1.357 (9)	C45—H45	0.9300
C4—H4	0.9300	C46—C47	1.353 (12)
C5—C6	1.376 (10)	C46—H46	0.9300
C5—C5^i^	1.499 (12)	C47—C48	1.379 (11)
C6—C7	1.366 (10)	C47—H47	0.9300
C6—H6	0.9300	C48—H48	0.9300
C7—H7	0.9300	C49—C50	1.385 (10)
C8—C9	1.385 (10)	C49—C54	1.389 (10)
C8—H8	0.9300	C50—C51	1.388 (11)
C9—C10	1.373 (10)	C50—H50	0.9300
C9—H9	0.9300	C51—C52	1.347 (14)
C10—C11	1.368 (10)	C51—H51	0.9300
C10—C10^ii^	1.477 (12)	C52—C53	1.369 (14)
C11—C12	1.370 (10)	C52—H52	0.9300
C11—H11	0.9300	C53—C54	1.408 (11)
C12—H12	0.9300	C53—H53	0.9300
C13—C18	1.385 (10)	C54—H54	0.9300
C13—C14	1.394 (9)	C55—C56	1.386 (11)
C14—C15	1.374 (12)	C55—C60	1.398 (11)
C14—H14	0.9300	C56—C57	1.411 (14)
C15—C16	1.337 (15)	C56—H56	0.9300
C15—H15	0.9300	C57—C58	1.365 (17)
C16—C17	1.366 (15)	C57—H57	0.9300
C16—H16	0.9300	C58—C59	1.342 (16)
C17—C18	1.406 (12)	C58—H58	0.9300
C17—H17	0.9300	C59—C60	1.383 (12)
C18—H18	0.9300	C59—H59	0.9300
C19—C20	1.380 (9)	C60—H60	0.9300
C19—C24	1.387 (9)	B1—F3	1.283 (15)
C20—C21	1.399 (10)	B1—F2	1.288 (15)
C20—H20	0.9300	B1—F4	1.329 (15)
C21—C22	1.386 (12)	B1—F1	1.444 (18)
C21—H21	0.9300	B2—F5	1.285 (12)
C22—C23	1.351 (12)	B2—F6	1.314 (14)
C22—H22	0.9300	B2—F8	1.352 (12)
C23—C24	1.392 (10)	B2—F7	1.366 (13)
C23—H23	0.9300	C61—Cl2	1.732 (10)
C24—H24	0.9300	C61—Cl1	1.751 (10)
C25—C30	1.375 (10)	C61—H61A	0.9700
C25—C26	1.382 (10)	C61—H61B	0.9700
C26—C27	1.373 (12)	C62—Cl3	1.728 (14)
C26—H26	0.9300	C62—Cl4	1.762 (14)
C27—C28	1.380 (14)	C62—H62A	0.9700
C27—H27	0.9300	C62—H62B	0.9700
C28—C29	1.346 (13)	C63—Cl5	1.708 (15)
C28—H28	0.9300	C63—Cl6	1.738 (14)
C29—C30	1.398 (11)	C63—H63A	0.9700
C29—H29	0.9300	C63—H63B	0.9700
			
N1—Cu1—P3	118.66 (17)	C28—C29—C30	120.5 (9)
N1—Cu1—P1	115.59 (17)	C28—C29—H29	119.7
P3—Cu1—P1	122.10 (7)	C30—C29—H29	119.7
N2—Cu2—P2	116.50 (18)	C25—C30—C29	120.2 (8)
N2—Cu2—P4	111.90 (18)	C25—C30—H30	119.9
P2—Cu2—P4	129.66 (7)	C29—C30—H30	119.9
C13—P1—C19	105.0 (3)	C32—C31—C36	117.6 (8)
C13—P1—C1	105.3 (3)	C32—C31—P2	119.0 (6)
C19—P1—C1	103.3 (3)	C36—C31—P2	123.4 (6)
C13—P1—Cu1	117.8 (2)	C31—C32—C33	121.5 (9)
C19—P1—Cu1	102.7 (2)	C31—C32—H32	119.2
C1—P1—Cu1	120.6 (2)	C33—C32—H32	119.2
C25—P2—C1	107.6 (3)	C34—C33—C32	120.4 (10)
C25—P2—C31	105.6 (3)	C34—C33—H33	119.8
C1—P2—C31	100.5 (3)	C32—C33—H33	119.8
C25—P2—Cu2	117.0 (2)	C35—C34—C33	118.2 (9)
C1—P2—Cu2	115.5 (2)	C35—C34—H34	120.9
C31—P2—Cu2	108.9 (2)	C33—C34—H34	120.9
C43—P3—C37	104.6 (3)	C34—C35—C36	121.0 (10)
C43—P3—C2	105.2 (3)	C34—C35—H35	119.5
C37—P3—C2	103.8 (3)	C36—C35—H35	119.5
C43—P3—Cu1	104.3 (2)	C31—C36—C35	121.3 (9)
C37—P3—Cu1	120.8 (2)	C31—C36—H36	119.4
C2—P3—Cu1	116.5 (2)	C35—C36—H36	119.4
C55—P4—C49	104.8 (3)	C38—C37—C42	118.6 (7)
C55—P4—C2	103.5 (3)	C38—C37—P3	120.4 (5)
C49—P4—C2	106.3 (3)	C42—C37—P3	121.0 (5)
C55—P4—Cu2	107.5 (2)	C37—C38—C39	121.3 (7)
C49—P4—Cu2	116.4 (2)	C37—C38—H38	119.4
C2—P4—Cu2	117.0 (2)	C39—C38—H38	119.4
C3—N1—C7	115.7 (6)	C40—C39—C38	120.1 (8)
C3—N1—Cu1	124.9 (5)	C40—C39—H39	120.0
C7—N1—Cu1	119.5 (5)	C38—C39—H39	120.0
C8—N2—C12	116.0 (6)	C39—C40—C41	119.8 (8)
C8—N2—Cu2	121.9 (5)	C39—C40—H40	120.1
C12—N2—Cu2	122.0 (5)	C41—C40—H40	120.1
P2—C1—P1	116.7 (3)	C40—C41—C42	120.5 (8)
P2—C1—H1A	108.1	C40—C41—H41	119.8
P1—C1—H1A	108.1	C42—C41—H41	119.8
P2—C1—H1B	108.1	C37—C42—C41	119.7 (7)
P1—C1—H1B	108.1	C37—C42—H42	120.1
H1A—C1—H1B	107.3	C41—C42—H42	120.1
P4—C2—P3	111.3 (3)	C44—C43—C48	117.9 (6)
P4—C2—H2A	109.4	C44—C43—P3	124.3 (5)
P3—C2—H2A	109.4	C48—C43—P3	117.7 (5)
P4—C2—H2B	109.4	C43—C44—C45	119.6 (8)
P3—C2—H2B	109.4	C43—C44—H44	120.2
H2A—C2—H2B	108.0	C45—C44—H44	120.2
N1—C3—C4	123.4 (7)	C46—C45—C44	121.1 (8)
N1—C3—H3	118.3	C46—C45—H45	119.4
C4—C3—H3	118.3	C44—C45—H45	119.4
C5—C4—C3	120.9 (7)	C47—C46—C45	119.8 (8)
C5—C4—H4	119.6	C47—C46—H46	120.1
C3—C4—H4	119.6	C45—C46—H46	120.1
C4—C5—C6	115.9 (6)	C46—C47—C48	120.5 (8)
C4—C5—C5^i^	123.0 (8)	C46—C47—H47	119.8
C6—C5—C5^i^	121.1 (8)	C48—C47—H47	119.8
C7—C6—C5	120.4 (7)	C47—C48—C43	121.1 (8)
C7—C6—H6	119.8	C47—C48—H48	119.5
C5—C6—H6	119.8	C43—C48—H48	119.5
N1—C7—C6	123.8 (7)	C50—C49—C54	118.6 (7)
N1—C7—H7	118.1	C50—C49—P4	119.4 (6)
C6—C7—H7	118.1	C54—C49—P4	122.0 (6)
N2—C8—C9	123.9 (8)	C49—C50—C51	120.5 (9)
N2—C8—H8	118.1	C49—C50—H50	119.8
C9—C8—H8	118.1	C51—C50—H50	119.8
C10—C9—C8	120.3 (8)	C52—C51—C50	120.9 (9)
C10—C9—H9	119.8	C52—C51—H51	119.5
C8—C9—H9	119.8	C50—C51—H51	119.5
C11—C10—C9	115.3 (6)	C51—C52—C53	120.1 (9)
C11—C10—C10^ii^	123.8 (8)	C51—C52—H52	119.9
C9—C10—C10^ii^	120.9 (8)	C53—C52—H52	119.9
C10—C11—C12	121.4 (7)	C52—C53—C54	120.2 (9)
C10—C11—H11	119.3	C52—C53—H53	119.9
C12—C11—H11	119.3	C54—C53—H53	119.9
N2—C12—C11	123.0 (7)	C49—C54—C53	119.6 (8)
N2—C12—H12	118.5	C49—C54—H54	120.2
C11—C12—H12	118.5	C53—C54—H54	120.2
C18—C13—C14	118.4 (7)	C56—C55—C60	117.6 (8)
C18—C13—P1	122.4 (6)	C56—C55—P4	118.8 (6)
C14—C13—P1	119.2 (6)	C60—C55—P4	123.1 (6)
C15—C14—C13	120.2 (9)	C55—C56—C57	119.0 (10)
C15—C14—H14	119.9	C55—C56—H56	120.5
C13—C14—H14	119.9	C57—C56—H56	120.5
C16—C15—C14	121.3 (9)	C58—C57—C56	121.0 (10)
C16—C15—H15	119.3	C58—C57—H57	119.5
C14—C15—H15	119.3	C56—C57—H57	119.5
C15—C16—C17	120.5 (9)	C59—C58—C57	120.5 (11)
C15—C16—H16	119.7	C59—C58—H58	119.7
C17—C16—H16	119.7	C57—C58—H58	119.7
C16—C17—C18	119.8 (10)	C58—C59—C60	119.7 (11)
C16—C17—H17	120.1	C58—C59—H59	120.2
C18—C17—H17	120.1	C60—C59—H59	120.2
C13—C18—C17	119.7 (9)	C59—C60—C55	122.1 (9)
C13—C18—H18	120.1	C59—C60—H60	119.0
C17—C18—H18	120.1	C55—C60—H60	119.0
C20—C19—C24	117.5 (6)	F3—B1—F2	113.8 (13)
C20—C19—P1	124.9 (5)	F3—B1—F4	112.0 (13)
C24—C19—P1	117.4 (5)	F2—B1—F4	117.5 (14)
C19—C20—C21	121.5 (7)	F3—B1—F1	112.6 (14)
C19—C20—H20	119.3	F2—B1—F1	98.0 (12)
C21—C20—H20	119.3	F4—B1—F1	101.4 (12)
C22—C21—C20	119.6 (8)	F5—B2—F6	112.8 (11)
C22—C21—H21	120.2	F5—B2—F8	111.8 (10)
C20—C21—H21	120.2	F6—B2—F8	111.0 (11)
C23—C22—C21	119.3 (7)	F5—B2—F7	110.1 (11)
C23—C22—H22	120.3	F6—B2—F7	99.9 (10)
C21—C22—H22	120.3	F8—B2—F7	110.7 (10)
C22—C23—C24	121.2 (8)	Cl2—C61—Cl1	116.6 (10)
C22—C23—H23	119.4	Cl2—C61—H61A	108.1
C24—C23—H23	119.4	Cl1—C61—H61A	108.1
C19—C24—C23	120.9 (7)	Cl2—C61—H61B	108.1
C19—C24—H24	119.5	Cl1—C61—H61B	108.1
C23—C24—H24	119.5	H61A—C61—H61B	107.3
C30—C25—C26	118.0 (7)	Cl3—C62—Cl4	109.8 (7)
C30—C25—P2	124.2 (5)	Cl3—C62—H62A	109.7
C26—C25—P2	117.8 (6)	Cl4—C62—H62A	109.7
C27—C26—C25	121.9 (9)	Cl3—C62—H62B	109.7
C27—C26—H26	119.1	Cl4—C62—H62B	109.7
C25—C26—H26	119.1	H62A—C62—H62B	108.2
C26—C27—C28	119.1 (8)	Cl5—C63—Cl6	110.0 (8)
C26—C27—H27	120.5	Cl5—C63—H63A	109.7
C28—C27—H27	120.5	Cl6—C63—H63A	109.7
C29—C28—C27	120.3 (8)	Cl5—C63—H63B	109.7
C29—C28—H28	119.9	Cl6—C63—H63B	109.7
C27—C28—H28	119.9	H63A—C63—H63B	108.2
			
N1—Cu1—P1—C13	−29.2 (3)	C21—C22—C23—C24	0.6 (13)
P3—Cu1—P1—C13	172.7 (2)	C20—C19—C24—C23	−0.4 (11)
N1—Cu1—P1—C19	85.5 (3)	P1—C19—C24—C23	174.5 (6)
P3—Cu1—P1—C19	−72.6 (2)	C22—C23—C24—C19	−0.4 (12)
N1—Cu1—P1—C1	−160.3 (3)	C1—P2—C25—C30	14.4 (7)
P3—Cu1—P1—C1	41.5 (2)	C31—P2—C25—C30	−92.2 (7)
N2—Cu2—P2—C25	16.8 (3)	Cu2—P2—C25—C30	146.4 (6)
P4—Cu2—P2—C25	179.5 (2)	C1—P2—C25—C26	−166.7 (6)
N2—Cu2—P2—C1	145.1 (3)	C31—P2—C25—C26	86.7 (6)
P4—Cu2—P2—C1	−52.2 (3)	Cu2—P2—C25—C26	−34.7 (7)
N2—Cu2—P2—C31	−102.8 (3)	C30—C25—C26—C27	−0.3 (12)
P4—Cu2—P2—C31	59.9 (2)	P2—C25—C26—C27	−179.3 (7)
N1—Cu1—P3—C43	−102.8 (3)	C25—C26—C27—C28	0.2 (14)
P1—Cu1—P3—C43	54.7 (2)	C26—C27—C28—C29	−0.4 (16)
N1—Cu1—P3—C37	14.2 (3)	C27—C28—C29—C30	0.7 (16)
P1—Cu1—P3—C37	171.8 (2)	C26—C25—C30—C29	0.6 (13)
N1—Cu1—P3—C2	141.7 (3)	P2—C25—C30—C29	179.5 (7)
P1—Cu1—P3—C2	−60.8 (2)	C28—C29—C30—C25	−0.9 (15)
N2—Cu2—P4—C55	72.7 (3)	C25—P2—C31—C32	−143.4 (8)
P2—Cu2—P4—C55	−90.6 (3)	C1—P2—C31—C32	104.9 (8)
N2—Cu2—P4—C49	−44.3 (3)	Cu2—P2—C31—C32	−16.9 (8)
P2—Cu2—P4—C49	152.4 (3)	C25—P2—C31—C36	40.8 (8)
N2—Cu2—P4—C2	−171.5 (3)	C1—P2—C31—C36	−71.0 (8)
P2—Cu2—P4—C2	25.2 (3)	Cu2—P2—C31—C36	167.3 (7)
P3—Cu1—N1—C3	67.7 (6)	C36—C31—C32—C33	1.0 (17)
P1—Cu1—N1—C3	−91.2 (6)	P2—C31—C32—C33	−175.1 (10)
P3—Cu1—N1—C7	−111.2 (6)	C31—C32—C33—C34	−2(2)
P1—Cu1—N1—C7	89.8 (7)	C32—C33—C34—C35	2(2)
P2—Cu2—N2—C8	−90.5 (6)	C33—C34—C35—C36	0(2)
P4—Cu2—N2—C8	103.8 (6)	C32—C31—C36—C35	0.5 (17)
P2—Cu2—N2—C12	93.0 (6)	P2—C31—C36—C35	176.4 (10)
P4—Cu2—N2—C12	−72.7 (6)	C34—C35—C36—C31	−1(2)
C25—P2—C1—P1	87.8 (4)	C43—P3—C37—C38	118.7 (6)
C31—P2—C1—P1	−162.0 (4)	C2—P3—C37—C38	−131.2 (6)
Cu2—P2—C1—P1	−45.0 (4)	Cu1—P3—C37—C38	1.8 (7)
C13—P1—C1—P2	−74.7 (4)	C43—P3—C37—C42	−62.0 (6)
C19—P1—C1—P2	175.5 (3)	C2—P3—C37—C42	48.1 (6)
Cu1—P1—C1—P2	61.7 (4)	Cu1—P3—C37—C42	−178.9 (5)
C55—P4—C2—P3	−167.7 (4)	C42—C37—C38—C39	0.5 (12)
C49—P4—C2—P3	−57.6 (4)	P3—C37—C38—C39	179.8 (6)
Cu2—P4—C2—P3	74.4 (4)	C37—C38—C39—C40	−0.7 (14)
C43—P3—C2—P4	−161.3 (3)	C38—C39—C40—C41	0.0 (14)
C37—P3—C2—P4	89.1 (4)	C39—C40—C41—C42	0.8 (14)
Cu1—P3—C2—P4	−46.3 (4)	C38—C37—C42—C41	0.3 (11)
C7—N1—C3—C4	−0.1 (11)	P3—C37—C42—C41	−179.0 (6)
Cu1—N1—C3—C4	−179.1 (6)	C40—C41—C42—C37	−1.0 (13)
N1—C3—C4—C5	0.4 (12)	C37—P3—C43—C44	116.2 (6)
C3—C4—C5—C6	−0.9 (11)	C2—P3—C43—C44	7.2 (7)
C3—C4—C5—C5^i^	−179.5 (8)	Cu1—P3—C43—C44	−116.0 (6)
C4—C5—C6—C7	1.2 (14)	C37—P3—C43—C48	−66.9 (6)
C5^i^—C5—C6—C7	179.8 (9)	C2—P3—C43—C48	−175.9 (5)
C3—N1—C7—C6	0.4 (14)	Cu1—P3—C43—C48	60.9 (5)
Cu1—N1—C7—C6	179.5 (8)	C48—C43—C44—C45	−0.9 (11)
C5—C6—C7—N1	−1.0 (17)	P3—C43—C44—C45	176.0 (6)
C12—N2—C8—C9	0.5 (13)	C43—C44—C45—C46	1.2 (13)
Cu2—N2—C8—C9	−176.2 (7)	C44—C45—C46—C47	−1.1 (14)
N2—C8—C9—C10	0.9 (15)	C45—C46—C47—C48	0.8 (13)
C8—C9—C10—C11	−2.4 (13)	C46—C47—C48—C43	−0.6 (12)
C8—C9—C10—C10^ii^	177.1 (9)	C44—C43—C48—C47	0.6 (10)
C9—C10—C11—C12	2.5 (12)	P3—C43—C48—C47	−176.5 (6)
C10^ii^—C10—C11—C12	−177.0 (9)	C55—P4—C49—C50	−134.3 (6)
C8—N2—C12—C11	−0.4 (12)	C2—P4—C49—C50	116.4 (6)
Cu2—N2—C12—C11	176.3 (6)	Cu2—P4—C49—C50	−15.8 (7)
C10—C11—C12—N2	−1.2 (13)	C55—P4—C49—C54	45.5 (7)
C19—P1—C13—C18	33.3 (7)	C2—P4—C49—C54	−63.7 (7)
C1—P1—C13—C18	−75.4 (6)	Cu2—P4—C49—C54	164.0 (5)
Cu1—P1—C13—C18	146.8 (5)	C54—C49—C50—C51	−0.1 (12)
C19—P1—C13—C14	−146.3 (6)	P4—C49—C50—C51	179.7 (7)
C1—P1—C13—C14	105.0 (6)	C49—C50—C51—C52	1.3 (15)
Cu1—P1—C13—C14	−32.8 (6)	C50—C51—C52—C53	−2.0 (17)
C18—C13—C14—C15	−0.4 (11)	C51—C52—C53—C54	1.6 (16)
P1—C13—C14—C15	179.1 (6)	C50—C49—C54—C53	−0.3 (12)
C13—C14—C15—C16	1.2 (14)	P4—C49—C54—C53	179.8 (6)
C14—C15—C16—C17	−0.7 (16)	C52—C53—C54—C49	−0.4 (14)
C15—C16—C17—C18	−0.5 (16)	C49—P4—C55—C56	52.9 (7)
C14—C13—C18—C17	−0.7 (11)	C2—P4—C55—C56	164.2 (7)
P1—C13—C18—C17	179.7 (7)	Cu2—P4—C55—C56	−71.4 (7)
C16—C17—C18—C13	1.2 (14)	C49—P4—C55—C60	−135.1 (7)
C13—P1—C19—C20	−116.5 (6)	C2—P4—C55—C60	−23.9 (7)
C1—P1—C19—C20	−6.4 (7)	Cu2—P4—C55—C60	100.6 (7)
Cu1—P1—C19—C20	119.8 (6)	C60—C55—C56—C57	−0.2 (13)
C13—P1—C19—C24	69.0 (6)	P4—C55—C56—C57	172.2 (8)
C1—P1—C19—C24	179.1 (5)	C55—C56—C57—C58	1.9 (18)
Cu1—P1—C19—C24	−54.7 (5)	C56—C57—C58—C59	−2(2)
C24—C19—C20—C21	1.1 (11)	C57—C58—C59—C60	0(2)
P1—C19—C20—C21	−173.4 (6)	C58—C59—C60—C55	2.2 (16)
C19—C20—C21—C22	−0.9 (13)	C56—C55—C60—C59	−1.8 (13)
C20—C21—C22—C23	0.0 (13)	P4—C55—C60—C59	−173.9 (8)

Symmetry codes: (i) −*x*+1, −*y*+1, −*z*; (ii) −*x*+1, −*y*+1, −*z*+1.
