# *catena*-Poly[[copper(I)-bis[μ-bis(di­phenyl­phos­phino)methane-κ^2^ *P*:*P*′]-copper(I)-μ-2,2′-bipyridine-κ^2^ *N*:*N*′] bis(tetra­fluorido­borate) dichloromethane 2.5-solvate]. Corrigendum

**DOI:** 10.1107/S160053680801711X

**Published:** 2008-06-21

**Authors:** Juan Mo, Gong-Zheng Hu, Wen Chen, Li Yuan, Yu-Shan Pan

**Affiliations:** a College of Animal Husbandry and Veterinary Studies, Henan Agricultural University, Zhengzhou, Henan Province 450002, People’s Republic of China

## Abstract

The title of the paper by Mo, Hu, Chen, Yuan & Pan [*Acta Cryst.* (2008), E**64**, m391] is corrected.

## Abstract 2

Corrigendum to *Acta Cryst.* (2008), E**64**, m391.

In the paper by Mo, Hu, Chen, Yuan & Pan [*Acta Cryst.* (2008), E**64**, m391], the correct title is ‘*catena*-Poly[[copper(I)-bis[μ-bis(di­phenyl­phos­phino)methane-κ^2^ *P*:*P*′]-copper(I)-μ-4,4′-bipyridine-κ^2^ *N*:*N*′] bis(tetra­fluorido­borate) dichloro­methane 2.5-solvate]’.

